# Replication of High Fetal Alcohol Spectrum Disorders Prevalence Rates, Child Characteristics, and Maternal Risk Factors in a Second Sample of Rural Communities in South Africa

**DOI:** 10.3390/ijerph14050522

**Published:** 2017-05-12

**Authors:** Philip A. May, Marlene M. De Vries, Anna-Susan Marais, Wendy O. Kalberg, David Buckley, Colleen M. Adnams, Julie M. Hasken, Barbara Tabachnick, Luther K. Robinson, Melanie A. Manning, Heidre Bezuidenhout, Margaret P. Adam, Kenneth L. Jones, Soraya Seedat, Charles D.H. Parry, H. Eugene Hoyme

**Affiliations:** 1 Nutrition Research Institute, University of North Carolina at Chapel Hill, Chapel Hill, NC 28081, USA; julie_hasken@unc.edu; 2 Department of Psychiatry, Stellenbosch University, Stellenbosch 7599, South Africa; mmdevries@sun.ac.za (M.M.D.V.); asmarais@sun.ac.za (A.-S.M.); heidreb@sun.ac.za (H.B.); sseedat@sun.ac.za (S.S.); Charles.Parry@mrc.ac.za (C.D.H.P.); 3 Center on Alcoholism, Substance Abuse and Addictions, University of New Mexico, Albuquerque, NM 87131, USA; wkalberg@unm.edu (W.O.K.); dbuckley@unm.edu (D.B.); 4 Department of Psychiatry and Mental Health, University of Cape Town, Cape Town 7700, South Africa; colleen.adnams@uct.ac.za; 5 Emerita of Psychology, California State University, Northridge, Los Angeles, CA 91330, USA; barbara.tabachnick@csun.edu; 6 Department of Pediatrics, State University of New York, Buffalo, NY 14222, USA; lutherkrobinson@gmail.com; 7 Departments of Pathology and Pediatrics, School of Medicine, Stanford University, Stanford, CA 94305, USA; mmanning@stanford.edu; 8 Department of Pediatrics, School of Medicine, University of Washington, Seattle, WA 98195, USA; Margaret.adam@seattlechildrens.org; 9 Department of Pediatrics, School of Medicine, University of California San Diego, La Jolla, CA 92093, USA; Klyons@ucsd.edu; 10 Alcohol, Tobacco, and Other Drug Research Unit, South African Medical Research Council, Cape Town 7501, South Africa; 11 Department of Pediatrics, University of South Dakota Sanford School of Medicine, Sioux Falls, SD 57105, USA; Gene.Hoyme@sanfordhealth.org; 12 Sanford Research, Sioux Falls, SD 57104, USA; 13 Department of Pediatrics, College of Medicine, University of Arizona, Tucson, AZ 85724, USA

**Keywords:** fetal alcohol spectrum disorders (FASD), microcephaly, prenatal alcohol use, binge drinking, alcohol abuse, maternal risk for FASD, prevalence, children with FASD, South Africa

## Abstract

*Background*: Prevalence and characteristics of fetal alcohol syndrome (FAS) and total fetal alcohol spectrum disorders (FASD) were studied in a second sample of three South African rural communities to assess change. *Methods*: Active case ascertainment focused on children with height, weight and/or head circumference ≤25th centile and randomly-selected children. Final diagnoses were based on dysmorphology, neurobehavioral scores, and maternal risk interviews. *Results*: Cardinal facial features, head circumference, and total dysmorphology scores differentiated specific FASD diagnostic categories in a somewhat linear fashion but all FASD traits were significantly worse than those of randomly-selected controls. Neurodevelopmental delays were significantly worse for children with FASD than controls. Binge alcohol use was clearly documented as the proximal maternal risk factor for FASD, and significant distal risk factors were: low body mass, education, and income; high gravidity, parity, and age at birth of the index child. FAS rates continue to extremely high in these communities at 89–129 per 1000 children. Total FASD affect 196–276 per 1000 or 20–28% of the children in these communities. *Conclusions*: Very high rates of FASD persist in these general populations where regular, heavy drinking, often in a binge fashion, co-occurs with low socioeconomic conditions.

## 1. Introduction

### 1.1. Diagnosing a Continuum

Fetal alcohol syndrome (FAS) was first defined by Jones and Smith in 1973 [1]. FAS is the phenotype and diagnosis for children with the most restricted growth, dysmorphia, and cognitive and behavioral impairments. Children with less consistent and less severe dysmorphia and growth impairment and who meet criteria for many of the phenotypic traits of FAS are diagnosed with one of three other specific diagnoses developed by a committee of the Institute of Medicine (IOM) and slightly revised since: FAS, partial FAS (PFAS), alcohol-related birth defects (ARBD), and alcohol-related neurodevelopmental disorder (ARND) [2,3,4]. These four diagnoses form a continuum, from most to least dysmorphic, and eventually have become known as fetal alcohol spectrum disorders (FASD) [5,6]. Although FASD may represent the most common cause of intellectual disability and the general concept is known to many, their diagnoses are frequently not made or assessed accurately (missed in 80% of cases or misdiagnosed in 7% of cases) [7]. Therefore the clinical literature is limited by a selective understanding that may only represent the children who are most dysmorphic or severely impaired. Because of this, FASD epidemiology information gathered through registries [8] or from standard clinical activities represent an incomplete understanding of the prevalence and characteristics of children with FASD, especially those with PFAS and ARND. Although FASD epidemiology studies are expensive to pursue and require cooperation and support of multiple constituencies [2], such studies add a broader perspective to the understanding of the variable nature of FASD risk factors and outcomes. FASD were believed for years to be rare, affecting an estimated maximum of 1% of the general population [9], but recent studies indicate that FASD prevalence is much higher than originally thought, ranging from 2% to 5% in the USA and Europe [10,11,12,13,14,15,16]. In certain subpopulations and in entire towns of the Republic of South Africa (ZA), FASD rates are much higher.

### 1.2. Epidemiology Studies of FASD in South Africa: Creating a Sampling Distribution

Communities studied to date in ZA have the highest reported general-population rates of FASD in the world [17]. From an epidemiologic perspective, ZA has proven to be an excellent venue for understanding the prevalence, characteristics, and general etiology of the FASD continuum. In one particular municipality, “Study Community One” (SC1), and surrounding rural areas in the Western Cape Province (WCP), five epidemiological samples of FASD prevalence and characteristics have been completed [18,19,20,21,22]. Repeated sampling of this one community created a sampling distribution for accurate period prevalence rates for this locale and for monitoring change over time. SC1 has been the site of much attention in the media and prevention and intervention activity over the past two decades. In the most recent sample in SC1, FAS affected 59–79 children per 1000, and total FASD rates were 170–233 per 1000 or 17% to 23% [21]. Furthermore, community studies carried out by other researchers in other communities of ZA [23,24,25,26] have also found similarly high rates of FAS and PFAS, although these studies do not diagnose ARND. Quite noteworthy, the prevalence of FASD is highest in rural areas surrounding small towns in most ZA studies. Previous study findings indicated that norms of regular binge drinking, low socioeconomic status (SES), insufficient nutrition, high fertility, and challenging conditions for child development combine to elevate the prevalence and severity of FASD in many ZA communities [27,28,29,30,31,32].

### 1.3. The Current Study

The sample presented here represents a second study of FASD (commenced in 2011) in a highly rural area with three small towns located one to two hours away from SC1. The study was initiated to assess whether there was change in the prevalence of FASD. The previous study in this region [33] included four towns (collectively called BRAM for study purposes) and their surrounding areas, and this study included three of the same towns (called BAR for this study). As differences between the four communities were negligible, the decision was made not to include the fourth community due to finances, scheduling limitations, and other constraints [33]. The BAR communities are more rural, remote, and characterized by lower SES than SC1. In the previous 2009 study of this region [33], rates of FASD were higher than any ever published, and the severity of cases was greater than documented in SC1. Predominantly low SES conditions and local norms of weekend and holiday binge drinking combined for greater challenges to fetal development and positive child outcomes than in SC1.

## 2. Materials and Methods

### 2.1. Sampling and Recruitment

As before [33], this study utilized active case ascertainment methodology implemented by a highly experienced multidisciplinary field research team and diagnostic team lead by pediatric dysmorphologists/medical geneticists and overseen by an epidemiologist. All protocols were reviewed and approved by the Ethics Committee of Stellenbosch University, Faculty of Medicine and Health Sciences and the IRB of the University of New Mexico (Medical School HRRC 96-209 and 00-422). Active, written consent for children to participate in the study was sought from parents and guardians of all first grade pupils (*n* = 1448) enrolled in all 32 primary schools of the three (BAR) communities; consent was received for 1083 (74.8%). Per ZA ethics requirements, written assent was also obtained from children seven years and older.

**Figure 1:**
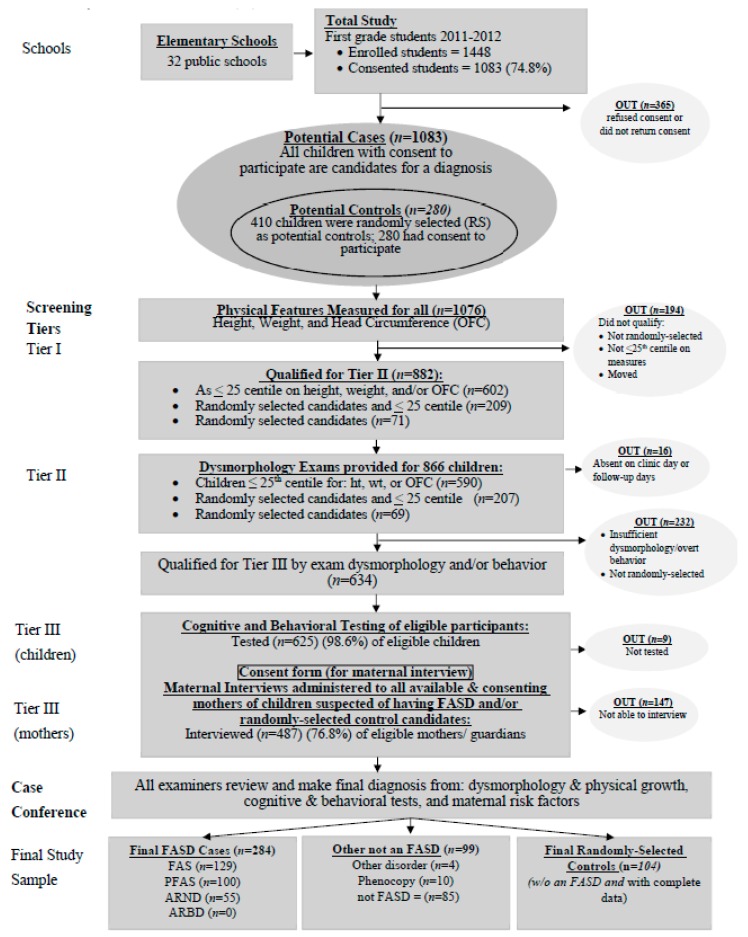
Sampling methodology for prevalence of FASD in three rural communities in a South African community: A second sample.

A three-tier process of screening, data collection, and diagnosis was instituted for all consented children (Figure 1). All consented children were measured for height, weight, and head circumference in Tier I. Each child who was ≤25th centile on height, weight, or occipitofrontal (head) circumference (OFC), was advanced to Tier II, an in-person pediatric dysmorphology exam. In addition, 410 child enrollment numbers were picked randomly from class lists as potential controls (normal/not FASD comparison children). Two-hundred-eighty (280) of the randomly selected children had consent to participate, and four did not complete all tiers of the study. Each qualifying child (small and/or randomly-selected) had 2-dimensional photographs taken and received the same dysmorphology exam from a dysmorphologist assisted by a scribe who recorded data on a standardized form. Of the children advanced to Tier III, the racial composition was a mirror of the 6-year olds of the region: 92.8% Coloured (mixed race), 5.4% Black, 1.6% White and 0.1% other.

### 2.2. IOM Diagnostic Categories

**Figure 2:**
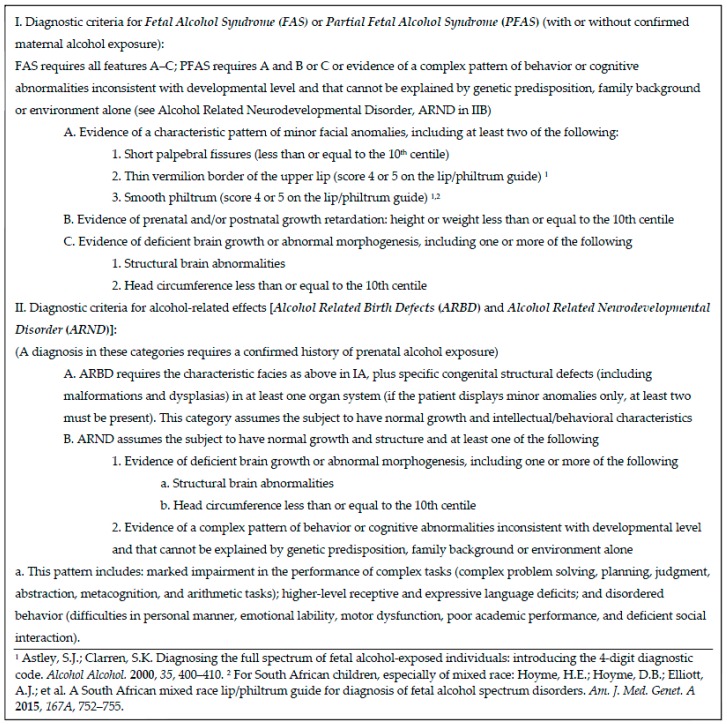
Diagnostic guidelines for specific fetal alcohol spectrum disorders (FASD), according to the Institute of Medicine, as clarified by Hoyme et al., 2005 [3].

IOM diagnostic criteria for FASD diagnoses are summarized in Figure 2 and described in detail elsewhere [4]. Two of the three cardinal features of FAS were assessed by the ZA lip/philtrum guide created especially for this population [34]. Significant growth retardation and specific dysmorphia must be present in children with FAS. Less growth restriction and slightly less dysmorphology is required in children with PFAS, but at least two of the three cardinal facial features and a constellation of other, specific minor anomalies must be present in both FAS and PFAS. The specific dysmorphology traits of FAS and PFAS have been clearly linked with prenatal alcohol exposure in thousands of cases, multiple epidemiology studies [21,33], and in correlation studies [25,29] where multiple confounders are controlled. Therefore, according to revised IOM guidelines, FAS and PFAS diagnoses can be made by qualified pediatricians without direct documentation of alcohol exposure after ruling out other malformation syndromes with similar phenotypes. In previous WCP studies, it has rarely been necessary to diagnose a child with FAS or PFAS without direct evidence of prenatal alcohol use [21,28,29,33,35].

For example, in the previous study of these communities, 88% of the mothers of children with FAS directly reported drinking during the index pregnancy [33]. Children with ARND do not have a characteristic pattern of facial characteristics, and therefore, direct evidence of prenatal alcohol exposure and significant cognitive impairment are explicitly required.

### 2.3. Assessment of Cognitive and Behavioral Traits

All randomly-selected control candidates and all children with significant traits common in a diagnosis within the continuum of FASD were advanced to cognitive testing in Tier III. Teachers completed Achenbach Teacher Report Forms (TRF) for each child [36] to characterize inattention and total behavioral problems. Cognitive tests utilized were: Test of Reception of Grammar (TROG) [37] for verbal ability; Raven Coloured Progressive Matrices [38] for non-verbal performance; and the Digit Span (subtest) of the Wechsler Intelligence Scales for Children, Third Edition [39] for working memory. Tests were administered in each child’s school by psychometrists blinded from any prior knowledge of the child or reason for testing. Tests were administered in Afrikaans (94%), the predominant regional language, 2% in English, and 4% in Xhosa, the most common Black African language of the region. Centile scores originate from standard charts of the Raven and TROG respectively. Scores falling at the 7th centile or below were 1.5 standard deviations below the sample mean. Scores reported for the Digit Span are scaled scores with a mean of 10 and standard deviation of 3. The two scores of the Achenbach (total problems and inattention) were interpreted as follows: total problem T score ≥64 fell in the clinical range; inattention T scores of ≥22 were in the clinical range.

### 2.4. Maternal Risk Factor Assessment: Proximal and Distal Variables

Also in Tier III, mothers of every child that could be located and consented were interviewed (96% in Afrikaans, 3% in Xhosa and 1% in English) regarding maternal risk for FASD in the index pregnancy. Time-line-follow-back methods [40,41] have been adapted for this population and used successfully in many ZA studies [20,21,27,32,33,42]. Proximal variables assessed were: alcohol use by quantity, frequency, and gestational timing and breastfeeding when also an active drinker. Distal risk variables were: maternal height, weight, and body mass index (BMI); childbearing history; demographic; and SES. General medical history was explored as the overarching theme, as was dietary intake, which facilitated cooperation with a questionnaire exploring alcohol and substance use [43]. As established previously in ZA studies, direct and presumably accurate maternal reports of prenatal alcohol use are obtained for a majority of respondents [44]. In this sample direct reports of any drinking during the index pregnancy were provided by: 84% of the mothers of children with FAS, 71% with PFAS, 100% with ARND, and 46% of controls.

### 2.5. Multidisciplinary Case Conference for Final Diagnosis

In multidisciplinary case conferences, findings from each domain (growth, dysmorphology, cognitive/behavioral performance, and maternal risk factors) were formally reviewed in oral presentations by the examiners of each child and interviewers of each mother. All other clinical team members listened while viewing photographs of each child projected on a screen to refresh everyone’s memory of the child. After review and discussion, final IOM criteria were reviewed and assessed, and a final diagnosis was made by the dysmorphologists with consensus of the entire clinical team.

### 2.6. Statistical Analysis

Case control analyses compare results across diagnostic groups and controls. Data were processed with Excel (Microsoft, Redmond, WA, USA) [45] and analyzed with SPSS 24 (IBM Co., Armonk, NY, USA) [46]. Statistical significance for categorical data utilized chi-square. For continuous data, one-way analysis of variance (ANOVA) was employed using Bonferroni-adjusted alpha values as indicated on each table [47]. With a statistically significant ANOVA, post-hoc analyses were performed using Dunnett’s correction pairwise comparisons (α = 0.05). We utilizes partial correlation analysis between maternal drinking during pregnancy and selected outcomes after adjusting for SES (income and mother’s education).

## 3. Results

### 3.1. Child Physical Growth and Development and Dysmorphology

**Figure 3:**
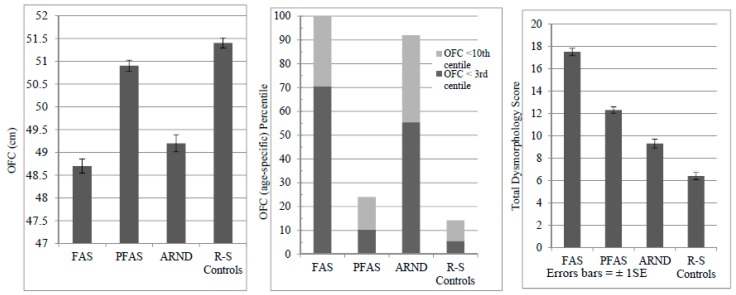
Average occipitofrontal (head) circumference **(**OFC) by measurement (cm) and age-specific percentile and total dysmorphology scores by diagnostic category for a second sample in three rural South African communities.

In Table 1, average values for the groups of children were correctly classified by IOM criteria, as children with FASD were significantly smaller than controls on height, weight, BMI, and head circumference (OFC), and demonstrated requisite cardinal features of FAS: reduced (short) palpebral fissure length (PFL), smooth philtrum, and narrow vermilion. Age and sex were not different across groups, indicating a tight age- and sex-specific cohort. Significant pairwise differences for individual physical traits across groups indicate that weight most distinguished the diagnostic groups. Also highly discriminating were: height, OFC, and PFL, as five of the pairwise comparisons were significantly different. OFC is most depressed among children with FAS (100%) and ARND (91%), mostly because of IOM criteria requiring OFC ≤10th centile for an FAS diagnosis, and all children with an OFC ≤10th centile were deferred by dysmorphologists to Tier III testing to rule out ARND. The PFAS diagnosis does not require a small head. Table 1 and Figure 3 indicate that microcephaly ≤3% existed in 70.5% of the FAS cases and in 56.4% of children with ARND.

Average total dysmorphology scores (Table 1) summarize FASD-relevant minor anomalies; scores are significantly different across groups, with highest scores in the FAS group (17.5), then PFAS (12.3), ARND (9.5), and finally controls (6.4). Variance is similar by group (SD = 2.9–3.7). Mean scores are linear across the spectrum and control group (Figure 3). In post-hoc analyses, each diagnostic group is significantly different from the other in total dysmorphology score. Even children with ARND have significantly more dysmorphology than controls.

Table 2 presents the results for other relevant minor anomalies that significantly differentiated the groups. Retarded growth measured in the maxillary and mandibular arcs were the most significant discriminators of the minor anomalies in Table 2 [48]. Short inner-canthal and inter-pupillary distance, hypoplastic midface, ptosis, camptodactyly of the fingers, and altered palmar creases were all significantly more common among FASD groups, and most common among children with FAS.

### 3.2. Cognitive and Behavioral Traits

Cognitive testing and behavioral checklist results (Table 3) characterize low mean achievement levels overall for children in this community, and ANOVA values indicate significant differences among all diagnostic groups. Mean values for the control group are best for all five measures, and ARND and FAS groups perform worst on each cognitive measure: verbal ability, non-verbal ability, and Digit Span. The ARND group has the highest average for behavioral and inattention problems; children with FAS have the next highest scores followed by PFAS. Post-hoc analysis indicates that non-verbal IQ and the Digit Span are the most discriminating measures.

### 3.3. Proximal Maternal Risk Variables: Alcohol Use during Pregnancy

Maternal alcohol consumption during pregnancy is detailed in Table 4. Significant differences exist among the groups for almost every variable. Forty-five percent of the mothers of normal controls reported drinking during the index pregnancies, and 55% abstained. The mothers of controls who drank were less likely to be frequent binge drinkers and drank the least in each trimester, especially in the second and third. Most mothers of controls who drank at all, commonly quit once pregnancy was clinically confirmed. The percentage of mothers of children with ARND who drank in each trimester was very high (1st = 100%, 2nd = 83%, and 3rd = 59%), but they generally drank fewer days per week and fewer binges of 5+ drinks than mothers of children with FAS. Average drinks per drinking day for the mothers of children with FAS was highest in every trimester (1st = 8.4, 2nd = 8.5, and 3rd = 8.9), and 61% drank in the third trimester. 

Seventy percent of mothers of children with PFAS, reported drinking in the index pregnancy, drinking averages of: 6.6, 6.7 to 7.5 drinks per drinking day during the respective trimesters; but they drank fewer days per week than mothers of FAS and ARND children. Among mothers of children with FASD, the PFAS group binged least in every trimester, drank less frequently, and was less likely to report drinking at all in 2nd and 3rd trimesters.

### 3.4. Correlating FASD Traits with Alcohol Use

Partial correlation analysis measured associations between maternal drinking and cognitive/behavioral measures, OFC, and total dysmorphology scores after adjusting for household income and mother’s education (Table 5). Transformations were undertaken for most measures due to skewness. Logarithmic transformations were applied to number of drinks per drinking day, average drinks per week, verbal IQ, and non-verbal IQ. Square root transformations were applied to behavior and inattention problems. Education was negatively skewed, so that scores were reflected before applying square root transformation. One extremely outlying score on income was recoded to become slightly greater than the next greatest score before square root transformation. Although highly unbalanced, transformations could not be applied to “yes/no” items: reported drinking during pregnancy and the two measures of binge drinking. A statistical criterion of *p* < 0.012 was set to control for Type I familywise error. The analysis included 432 children for whom mothers’ education and family income were reported.

Controlling for two measures of socioeconomic status (SES), all three measures of drinking during pregnancy (drank during pregnancy, drinks per drinking day, and drinks per week) correlated significantly with all cognitive and behavioral scores using Bonferroni-adjusted values. Verbal and non-verbal IQ and Digit Span performance were significantly lower with prenatal drinking. Behavior problems and inattention were greater with prenatal drinking. However, none of these partial correlations were particularly strong once adjusted for SES, with *r* ranging from −0.134 to −0.219. Thus, each drinking variable accounts for only about 3% of the variance in the child’s cognitive or behavioral scores. Binge drinking measures of drinks per drinking day and 3+ and 5+ drinks per occasion correlated most with head circumference and accounted for 5–6% of the variance in head size. More drinking is associated with smaller head size. Behavioral measures correlated in the expected direction, but not with cognitive measures. Again, the signs of the correlations were all in the expected direction, but were not particularly strong.

Maternal drinking measures correlated most highly with the child’s OFC after adjusting for income and education: with partial *r* ranging from 0.206 to 0.256. Prenatal drinking was significantly associated with total dysmorphology score as well (*r* ranging from 0.168 to 0.215). No statistically significant associations were noted between paternal drinking problems and child traits after adjusting for socioeconomic measures and mothers’ weekly quantity of drinking during pregnancy.

### 3.5. Tobacco Use

A significantly higher percentage of FASD mothers than non-FASD mothers reported smoking during the index pregnancy (Table 4). The quantity of cigarette consumption by smokers did not differ across groups. A high percentage of women smoke, but average weekly quantity is low (<20 g). Use of drugs other than alcohol was not significantly different among groups during pregnancy or lifetime. Alcohol is the primary teratogen.

### 3.6. Distal Maternal Risk Traits—Physical, Childbearing, and Demographic

In Table 4, significant distal maternal risk variables are: older age at birth of the index child, lower weight, low BMI, and less educational achievement. High gravidity and parity are also more common for mothers of children with FASD. Rural residence did not differ significantly across groups even though over 46% of all children with FASD underwent gestation there.

### 3.7. Prevalence Estimates by Three Methods, Their Calculation, and the Final Estimates

Final diagnoses of children and the first estimation of prevalence are presented in the left side of Table 6. There were 129 children diagnosed with FAS, 100 with PFAS, and 55 with ARND. No cases of ARBD were found. With the first estimation technique, two different denominators were used: children enrolled in all community 1st grade classes (*n* = 1448) for the low estimate, and the total number with consent (*n* = 1088) for the upper estimate. Oversampling of small children provided the greatest probability of including virtually every child with FAS or PFAS. The rate of FAS with this technique is 89.1–118.6 per 1000, and the rate of total FASD is 196.1–261.0 per 1000.

**Figure 4:**
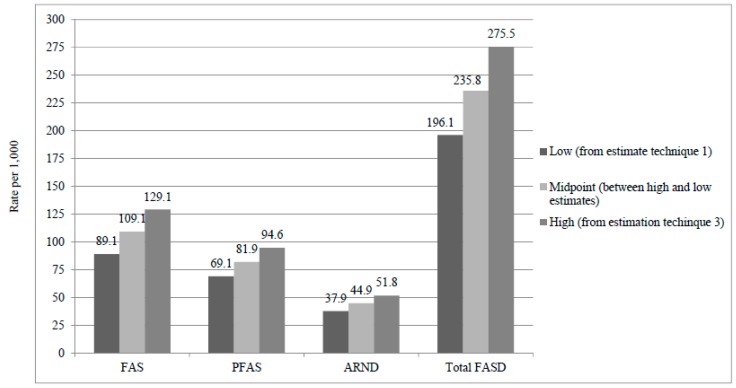
Final prevalence rates (per 1000) of FASD diagnostic groups and controls in three rural communities South Africa: A second sample.

A second rate was calculated from the 87 cases of FASD found within the 276 children who entered the study via random selection as candidates for the control group (middle section, Table 6). FAS and total FASD rates from this technique are: 159.4 FAS cases per 1000 (95% CI = 116.2 to 202.6) and total FASD rate is 315.2 per 1000 (95% CI = 260.4 to 370.0) with this technique. This rate is the highest, in part because the random sample was relatively small, and because of the small sample we were less confident that this is the most accurate rate. It is therefore not presented in Figure 4.

The third set of rates (Table 6, right section) was calculated by estimating the total number of cases which would likely exist among the unconsented children and adding these estimated cases to cases diagnosed among consented children. Using the proportions of individual and total FASD diagnoses in the random sample (total FASD = 0.3152) to estimate the number of FASD cases among the 365 unconsented children (b = 115) and through adding them to the FASD cases diagnosed (a = 284) in the consented population (a + b = 399), technique 3 estimates FAS to be 129.1 per 1000 and total FASD to be 275.5 per 1000 (95% CI = 252.6 to 298.6 per 1000). We feel this is the most accurate estimate of the high estimate in a range of rates, and it is used as the higher rate in Figure 4.

### 3.8. Comparing Sample 1 to Sample 2 in This Region

The first sample in this region commenced in 2009 and data collection lasted for two years. The second sample skipped a school year and commenced with the first graders enrolled in 2011. Table 7 compares the final range of rates over the two different time periods. Rates are similar for most every diagnosis over time. Case ratios for individual diagnostic categories are also virtually identical.

## 4. Discussion

### 4.1. Summary of Findings

The physical, cognitive, and behavioral traits of children with FASD in these rural, low SES communities are virtually identical to those of the previous sample [33]. Child growth, development, and most minor anomalies were clearly categorized in a somewhat linear fashion by the revised IOM criteria, the only exceptions were that on many neurobehavioral traits/variables, children with FAS and ARND were more impaired than were the children with PFAS. FASD prevalence continues to be higher in these more rural areas (20–27%) than in recent samples of the more urban, SC1 (17–23%) [21]. This research has once again empirically linked FASD traits to detailed self-reporting of prenatal alcohol use in the index pregnancy, even when SES is controlled.

Additionally, many distal variables of maternal risk in this sample were similar to the previous study in these communities [33], and in most previous studies in the WCP. Less than optimal maternal health factors were linked to the FASD cases: low BMI and low SES coupled with high gravidity and parity, advanced maternal age, and other challenging prenatal, postpartum, and environmental conditions for child growth and development. Outcomes, both physical and cognitive/behavioral, are especially poor among children who were exposed to the highest quantity and frequency of drinking, especially drinks per drinking day and three or more drinks per occasion in both the case control comparisons and the correlation analysis. Those who underwent gestation in low SES, rural conditions had the worst or more severe outcomes.

### 4.2. Head Circumference Is Suppressed in Children with FASD

Children with FAS and ARND had the smallest head circumferences and performed the worst on cognitive and behavioral assessments. Furthermore, the correlation analysis linked alcohol use in the prenatal period more clearly to small head circumference than any other variable. Prenatal drinking is the most common cause of microcephaly in these communities, and a greater recognition of the teratogenic properties of alcohol is needed by public health researchers and professionals around the world. While current concerns over microcephaly from Zika virus are appropriate, ignoring the high prevalence of suppressed head growth and microcephaly from prenatal alcohol exposure is a continuing public health oversight. More cases of microcephaly and suppression of brain growth are currently caused by substantial prenatal alcohol exposure than by the Zika virus or other known agents.

### 4.3. Severity: Almost Half of All FASD Diagnosed in This Sample Meet Criteria for FAS

The severity of FASD in this region is greater than found in populations in the United States [12] and Italy [11]. FAS prevalence (the most severe form of FASD) is again found to be more common than any other individual diagnosis in the FASD continuum. We found that 51% of the FASD cases diagnosed in this region were FAS in sample 1 (2009) and 45% in this sample. The case ratio is 0.7 PFAS to 1 FAS, 0.4 ARND to 1 FAS in both samples, and overall, there were 1.0 to 1.1 other FASD cases to each case of FAS (Table 7). In a study with identical methodology in the United States, 18.6% of the total FASD cases were FAS, and case ratios were: 1.9 PFAS and 1.6 ARND to 1 FAS. In Italy there were 4.5 cases of PFAS to each case of FAS. This level of severity in ZA is most likely due to prevalent and regular weekend binge drinking in these communities, other distal variables of high risk, and possibly some exacerbation from alcohol consumption during the extended period of breastfeeding. In an analysis from previous studies in the WCP: the average period of breastfeeding was 18–19 months, 71% of all mothers reported drinking during the period of breastfeeding, and among mothers of children with FAS, PFAS, and ARND, it was 92%, 82% and 87% respectively [49]. Women in the USA and Italy have lower rates of breastfeeding overall. Also, mothers and children in the USA and Italy experience lower fertility and more favorable health, nutrition, BMI, and SES conditions which likely moderate or minimizes severity [12]. Conversely, virtually all mothers in the study communities of the WCP are undernourished [30,31] which is linked to depressed growth, development, and head circumference when prenatal alcohol exposure occurs. Whether rural residence during gestation is a primary or secondary risk factor is unknown. Does alcohol produce more damage in rural children due to more poverty, high fertility, and particularly rustic conditions, or does it reflect a more risky modal drinking pattern in both the prenatal period and through additional alcohol delivered via breastfeeding? We believe that each of these factors plays some role in the higher rates of FASD and greater representation of children with full FAS.

### 4.4. Convergence of Prevalence Rates from Estimation Techniques and Stability of Rates

Prevalence estimates from three common techniques again converge in this sample [12,21,33]. The highest rates were from the random sample alone (technique 2) where total FASD is 315 per 1000 (or 32%) with 95% confidence intervals of 260 to 370 per 1000 children. This is an outlier from the rates of the other two methods, and it is best not to estimate prevalence from this relatively smaller sample (even though it was random). Technique 1 data are the most empirical and robust, for 75% of all children in this population, especially those who are small (and most likely to have a FASD), have been screened and examined thoroughly. Technique 1 cases provide a very conservative, but highly empirical, low rate when coupled with the denominator for all enrolled students. Technique 3 is the most reliable high estimate because it combines the diagnosed cases from the consented sample with cases estimated from random-sample-derived proportions which are projected to the non-consented children. Therefore, the final rates that we present (Figure 4 and Table 6) were produced from a careful consideration of the most empirically-driven clinical assessments and random-sampling driven proportions from the various techniques.

The final prevalence findings between the first and second samples in this region, drawn two years apart are both similar and stable over this short period of time. The final rates in this second sample are: FAS, 89–129 per 1000, and total FASD, 196–276 per 1000 or 20–28%. This compares to the previous study where FAS was 93–128 per 1000, and total FASD was 182–259 per 1000 or 18–26%.

### 4.5. Linking FASD to Prenatal Alcohol Use

All individual parts of the study (dysmorphology exams, testing, and maternal interviews) were performed blinded by professionals in their individual disciplines; therefore, linking each domain for the final diagnosis is important. Furthermore, it is vital to link the traits and diagnoses of the children to the quantity, frequency, and gestational timing of maternal alcohol use in the prenatal period. This was accomplished by direct maternal reports in 87% of the FASD cases and in the remaining cases by collateral reports from relatives, neighbors, and other knowledgeable individuals. Both case control and correlation analyses linked drinking to child traits: more drinking during the index pregnancy led to suppressed growth and physical development, more minor anomalies, and poorer performance on cognitive and behavioral measures. The latter association of drinking and cognitive performance, while significant, is not as strong as the link with microcephaly and dysmorphology overall, has also been demonstrated with complex multivariate structural models [35,50]. Dysmorphology correlates most highly with drinking. The distal maternal risk variables studied once again correlate with additional individual variation in child outcomes in this sample, more variance than correlates with alcohol use alone. As evidenced in the FASD maternal groups, if a mother is on the wrong end of the distal maternal risk variables, severity of poor child outcomes is increased.

### 4.6. Limitations

This multiple-method, multi-disciplinary, comprehensive study of FASD in the general population of three communities had limitations. First, since the populations of these communities are substantially unique in the world, extrapolating directly from the exact findings, measures, and values for most variables should only be done with caution. Second, maternal interviews for the index pregnancies were administered to the mothers seven years post-partum. Recall may have been negatively affected for some variables, although several studies show otherwise [51,52]. Third, basing much of the initial study sample on child growth and development and dysmorphology, the prevalence of ARND is likely under-evaluated and under-estimated. However, an exception to this lower ARND rate is found in the random-selection rates for FASD in technique #2, for the rates do not reflect any screening for size. Fourth, administering a more extensive battery of cognitive and behavioral instruments would have been desirable, but this was well beyond the scope, time, and resources of this large epidemiology study. Finally, without accurate alcohol biomarker samples administered to the interviewed mothers, it is impossible to know exactly how precise and accurate the reporting is. Pilot examinations with two alcohol-use biomarkers have concluded that women from these communities are indeed quite honest in their reporting [44].

## 5. Conclusions

The rates of FASD in these towns and surrounding rural areas were stable between samples collected two years apart and remain higher than reported in a general population anywhere. Recreational binge drinking on the weekends is the primary, proximal risk factor for FASD, and a very high prevalence of FASD results. The traits of children with FAS, PFAS and ARND are clearly different from one another and from normal controls on most every measure. Furthermore, multiple distal maternal risk factors exist to varying degrees across this population, and the presence or absence of many of these factors differentiates the severity of child outcomes across groups. The rate of FAS remains extremely high, at 89–129 per 1000. The rates of PFAS and ARND are again high and similar to the previous sample. The total FASD rate of 20–28% in this sample represents the highest prevalence ever reported for a general population.

## Figures and Tables

**Table 1:** Children’s demographic, growth, cardinal fetal alcohol spectrum disorders (FASD) variables, and total dysmorphology score from three rural communities in South Africa with post hoc analysis summary.

	All Children ^1^	Children with FAS (*n* = 129)		Children with Partial FAS (*n* = 100)		Children with ARND (*n* = 55)		Randomly-Selected Normal Controls (*n* = 104)		Statistical Test	*p*
Sex (% male)	53.6	52.7		44.0		52.7		54.3		χ^2^ = 2.630	0.452
Age (months)—Mean (Standard Deviation (SD))	84.2 (9.1)	86.5	(10.1)	85.1	(8.7)	85.4	(9.0)	83.6	(10.7)	*F* = 1.644	0.179
Height (cm)—Mean (SD)	118.3 (20.6)	112.0	(5.9)	116.1	(6.0)	114.7	(4.8)	118.4	(6.0)	*F* = 52.681	<0.001 ^a,b,c,e,f^
Weight (kg)—Mean (SD)	17.2 (21.2)	17.5	(2.7)	19.9	(2.8)	18.5	(2.5)	21.3	(4.0)	*F* = 43.675	<0.001 ^a,b,c,d,e,f^
Child’s BMI—Mean (SD)	14.6 (1.6)	13.9	(1.3)	14.7	(1.2)	14.3	(2.6)	15.1	(1.8)	*F* = 10.947	<0.001 ^a,c^
BMI Percentile—Mean (SD)	27.3 (25.5)	15.0	(20.1)	29.9	(25.4)	16.3	(18.8)	35.8	(28.3)	*F* = 18.590	<0.001 ^a,c,d,f^
OFC (cm)—Mean (SD)	--	48.7	(1.2)	50.9	(1.2)	49.1	(1.2)	51.4	(1.4)	*F* = 91.713	<0.001 ^a,c,d,e,f^
<3rd centile (%)	--	70.5		10.0		56.4		5.7		*F* = 148.766	<0.001
<10th centile (%)	--	100.0		24.0		92.7		14.3		*F* = 247.530	<0.001
PFL centile—Mean (SD)	--	7.3	(11.6)	13.6	(15.3)	25.7	(14.3)	27.6	(15.5)	*F* = 48.925	<0.001 ^a,b,c,d,e^
Smooth Philtrum ^2^ (%)	--	76.7		79.0		18.2		26.7		χ^2^ = 112.305	<0.001
Narrow Vermilion ^2^ (%)	--	87.6		80.0		18.2		19.0		χ^2^ = 166.675	<0.001
Total Dysmorphology Score—Mean (SD)	--	17.5	(3.7)	12.3	(2.9)	9.5	(3.1)	6.4	(3.3)	*F* = 225.696	<0.001 ^a,b,c,d,e,f^

^1^ The “All Children” group is not included in any of the Table 1 statistical test analyses. ^2^ Scores of 4 or 5 on Astley Lip Philtrum Guide. Bonferroni-adjusted value: *p* < 0.0045. Significant (*p* < 0.05) post-hoc Dunnett C comparisons between: ^a^ fetal alcohol syndrome (FAS) & partial fetal alcohol syndrome (PFAS); ^b^ FAS & alcohol-related neurobehavioral disorder (ARND); ^c^ FAS & Controls; ^d^ PFAS & ARND; ^e^ PFAS & Controls ^f^ ARND & Controls.

**Table 2:** Other minor anomalies of children with FAS, PFAS, and ARND compared to controls from three rural communities in South Africa.

	Children with FAS (*n* = 129)	Children with Partial FAS (*n* = 100)	Children with ARND (*n* = 55)	Randomly-Selected Normal Controls (*n* = 104)	Test Score	*p* ^f^
Maxillary Arc (cm)	23.1 (1.2)	23.9 (0.9)	23.6 (0.9)	24.1 (1.3)	*F* = 19.187	<0.001 ^a,b,c,d,e^
Mandibular Arc (cm)	24.1 (1.3)	25.1 (1.0)	24.6 (1.1)	25.2 (1.5)	*F* = 17.951	<0.001 ^a,c,d,e^
Inner canthal distance (centile)	53.7 (22.5)	59.3 (20.6)	51.9 (19.7)	60.0 (22.9)	*F =* 2.950	<0.001
Inter-pupillary distance (centile)	44.7 (25.1)	54.1 (25.2)	53.1 (26.0)	58.5 (25.9)	*F =* 5.992	<0.001 ^a,c^
Hypoplastic midface (%)	76.7	61.0	41.8	20.8	χ^2^ = 31.398	<0.001
“Railroad” track ears (%)	53.3	20.0	13.3	13.3	χ^2^ = 10.429	0.015
Ptosis (%)	17.8	5.1	3.6	2.9	χ^2^ = 21.911	<0.001
Camptodactyly (%)	22.5	3.0	5.5	5.7	χ^2^ = 29.638	<0.001
Altered palmar creases (%)	45.7	32.0	27.3	22.9	χ^2^ = 15.084	0.002
Anteverted nostrils (%)	19.4	24.0	3.6	14.3	χ^2^ = 11.518	0.009

Significant (*p* < 0.05) post-hoc Dunnett C comparisons between: ^a^ FAS & PFAS; ^b^ FAS & ARND; ^c^ FAS & Controls; ^d^ PFAS & ARND; ^e^ ARND & Controls; ^f^ Bonferroni-adjusted value: *p* < 0.005.

**Table 3:** Mean scores on developmental and behavioral indicators ^1^ of children with FAS, PFAS, and ARND compared to controls from three rural communities in South Africa with post hoc analyses.

	Children with FAS (*n* = 125)		Children with Partial FAS (*n* = 99)		Children with ARND (*n* = 55)		Randomly-Selected Normal Controls (*n* = 101)		*F*	*p*
	Mean	(SD)	Mean	(SD)	Mean	(SD)	Mean	(SD)		
Verbal Ability ^^^ (percentile scores)	9.0	(11.6)	13.6	(18.1)	8.3	(11.6)	23.6	(21.2)	*F =* 17.567	<0.001 ^b,d,e^
Non-verbal Ability ^+^ (percentile scores)	12.6	(12.8)	20.0	(18.2)	10.9	(7.6)	28.1	(23.1)	*F* = 19.691	<0.001 ^a,b,c,d,e^
WISC-IV Digit-Span Scaled Score ^†^	4.6	(2.8)	5.7	(2.8)	4.3	(2.7)	6.9	(2.7)	*F* = 16.139	<0.001 ^a,b,c,d,e^
Teacher Report Form (TRF) Total Problem Score	34.5	(24.9)	29.8	(25.7)	41.9	(32.2)	19.9	(19.5)	*F* = 11.063	<0.001 ^b,d,e^
TRF Inattention Score	17.9	(12.0)	14.2	(11.5)	18.9	(12.3)	9.7	(8.9)	*F* = 12.807	<0.001 ^b,d,e^

^1^ All scores standardized for age of child at time of testing. ^^^ Test of Reception of Grammar (TROG). A measure of verbal intelligence. ^+^ Raven Coloured Progressive Matrices. A measure of nonverbal intelligence. ^†^ WISC-IV Digit Span Scaled Score—mean of 10 and standard deviation of 3. Bonferroni-adjusted value: *p* < 0.01; Significant (*p* < 0.05) post-hoc Dunnett C comparisons between: ^a^ FAS & PFAS; ^b^ FAS & Controls; ^c^ PFAS & ARND; ^d^ PFAS & Controls; ^e^ ARND & Controls.

**Table 4:** Maternal demographic, childbearing, socioeconomic, drinking, tobacco, and other drug use from three rural communities in South Africa: Mothers of children with FASD and normal controls.

	Mothers of									Statistical Test	*p*
	Children with FAS (*n* = 118)		Children with Partial FAS (*n* = 91)		Children with ARND (*n* = 55)		Randomly-Selected Normal Controls (*n* = 100)				
**Alcohol Consumption Variables**											
Current drinker (% Yes)	50.8		34.1		52.9		27.6			χ^2^ = 16.829	<0.001
Drank before index pregnancy (% Yes)	84.6		69.1		100.0		47.0			χ^2^ = 63.450	<0.001
Drank during index pregnancy (% Yes)	84.9		69.8		100.0		45.0			χ^2^ = 69.170	<0.001
Average # drinks per day during pregnancy	5.9 (5.8)		2.9 (4.3)		6.1 (5.9)		1.7 (3.4)			*F* = 17.599	<0.001 ^a,c,d,f^
Average # of drinking days during pregnancy	1.7 (1.6)		0.8 (1.0)		1.5 (1.4)		0.4 (0.7)			*F* = 23.910	<0.001 ^a,c,d,e,f^
Consumed **3 drinks** or more per occasion during pregnancy (%) ^	73.7		53.8		94.2		34.0			χ^2^ = 64.450	<0.001
Consumed **5 drinks** or more per occasion during pregnancy (%) ^	65.3		39.6		57.7		26.0			χ^2^ = 37.909	<0.001
**Alcohol Use by Trimester**											
Drank during **1st trimester** (% Yes)	81.0		67.0		100.0		43.0			χ^2^ = 66.045	<0.001
Binged 3+ (%) ^	72.2		53.8		92.2		31.3			χ^2^ = 63.795	<0.001
Binged 5+ (%) ^	63.5		38.5		54.9		23.2			χ^2^ = 38.449	<0.001
Average # of drinks per drinking day ^1^	8.4 (5.4)		6.6 (4.8)		7.9 (7.1)		5.7 (3.9)			*F* = 3.655	0.013 ^c^
# of drinking days per week ^1^	2.4 (1.5)		1.8 (1.2)		1.8 (1.3)		1.5 (0.7)			*F* = 6.087	0.001 ^a,c^
Drank during **2nd trimester** (% Yes)	71.4		45.2		83.3		25.0			χ^2^ = 70.927	<0.001
Binged 3+ (%) ^	58.3		35.2		74.5		18.2			χ^2^ = 58.529	<0.001
Binged 5+ (%) ^	53.0		25.3		41.2		14.1			χ^2^ = 40.526	<0.001
Average # of drinks per drinking day ^1^	8.5 (5.7)		6.7 (5.3)		7.4 (6.3)		6.6 (4.8)			*F* = 1.241	0.296
# of drinking days per week ^1^	2.4 (1.4)		1.8 (1.4)		1.9 (1.4)		1.7 (0.8)			*F* = 2.955	0.034 ^c^
Drank during **3rd trimester** (% Yes)	60.6		24.5		59.3		13.1			χ^2^ = 70.627	<0.001
Binged 3+ (%) ^	50.9		16.5		47.1		11.0			χ^2^ = 55.713	<0.001
Binged 5+ (%) ^	44.8		12.1		29.4		10.0			χ^2^ = 45.421	<0.001
Average # of drinks per drinking day ^1^	8.9 (5.5)		7.5 (6.8)		7.3 (6.6)		8.1 (5.6)			*F* = 0.687	0.562
# of drinking days per week ^1^	2.5 (1.5)		1.7 (1.4)		2.1 (1.6)		2.0 (0.7)			*F* = 2.325	0.078
**Tobacco and Use of Other Drugs**											
Other Drug Use in lifetime (%)	7.2		11.6		7.4		12.0			χ^2^ = 2.162	0.529
Other Drug Use during pregnancy (%)	2.5		5.4		1.8		2.1			χ^2^ = 2.470	0.481
Used tobacco during index pregnancy (%)	75.0		53.2		65.5		32.7			χ^2^ = 42.221	<0.001
Current smoker, smoked within week (%)	83.9		70.2		76.7		15.9			χ^2^ = 4.125	0.248
Total # of grams of tobacco used per week (each cigarette =1 g)	19.1 (20.9)		16.8 (29.3)		17.3 (22.0)		19.1 (31.8)			*F* = 0.180	0.910
**Demographics**											
Age at pregnancy (year)—Mean (SD)	29.1	(6.4)	27.1	(7.2)	26.0	(6.9)		25.1	(7.3)	*F* = 6.186	<0.001 ^b,c^
Height (cm)—Mean (SD)	155.2	(6.2)	156.6	(6.1)	157.8	(7.9)		158.1	(6.5)	*F* = 3.682	0.012 ^c^
Weight (kg)—Mean (SD)	58.9	(17.1)	66.7	(16.5)	64.2	(15.8)		73.4	(17.5)	*F* = 12.518	<0.001 ^a,c,f^
Body Mass Index (BMI)—Mean (SD)	24.4	(6.9)	27.2	(6.8)	25.7	(6.4)		29.4	(6.8)	*F* = 9.419	<0.001 ^a,c,f^
Occipitofrontal circumference (OFC)—Mean (SD)	54.7	(2.1)	55.0	(1.9)	55.0	(1.6)		55.5	(2.0)	*F* = 3.005	0.031 ^c^
Gravidity—Mean (SD)	3.8	(1.6)	3.3	(1.7)	3.1	(1.5)		2.9	(1.4)	*F* = 6.355	<0.001 ^b,c^
Parity—Mean (SD)	3.3	(1.5)	3.1	(1.6)	2.7	(1.2)		2.6	(1.4)	*F* = 5.202	0.002 ^b,c^
Miscarriages—Mean (SD)	0.3	(0.7)	0.2	(0.5)	0.2	(0.5)		0.2	(0.4)	*F* = 1.710	0.164
Stillbirths—Mean (SD)	0.09	(0.3)	0.04	(0.5)	0.05	(0.2)		0.06	(0.3)	*F* = 0.557	0.644
Breastfed index child (% Yes)	89.0		93.5		81.8		88.8			χ^2^ = 4.780	0.189
Duration of breastfeeding (months)—Mean (SD)	20.2	(19.2)	20.3	(21.3)	22.4	(21.4)		21.5	(19.8)	*F* = 0.180	0.910
Maternal education (years)—Mean (SD)	6.8	(3.4)	8.1	(3.1)	8.6	(3.2)		9.3	(2.7)	*F* = 11.771	<0.001 ^a,b,c,e^
Residence during pregnancy (% Rural)	51.3		49.5		45.5		32.0			χ^2^ = 9.463	0.024
Income (Rand per week)—Mean (SD)	818	(586)	945	(546)	829	(534)		1406	(2669)	*F* = 3.349	0.019

^1^ Drinkers only for that trimester. ^a^ FAS & PFAS; ^b^ FAS & ARND; ^c^ FAS & Controls; ^d^ PFAS & ARND; ^e^ PFAS & Controls; ^f^ ARND & Controls. Bonferroni-adjusted values by Table section: alcohol consumption *p* < 0.007; alcohol by trimester *p* < 0.003; tobacco and other drugs *p* < 0.01; demographics *p* < 0.004. ^ Excludes women who collaterals confirmed alcohol consumption during pregnancy, but did not know quantity of consumption.

**Table 5:** Partial correlation coefficients (adjusted for square root of household income and square root of mother’s education) for developmental ^1^ and physical dysmorphology variables with selected maternal drinking measures during pregnancy and paternal drinking from three rural communities in South Africa.

Child Trait		Mothers Reported Drinking during Pregnancy	Drinks per Drinking Day during Pregnancy (log)	Drinks per Week during Pregnancy (log)	3 or More Drinks per Occasion during Pregnancy	5 or More Drinks per Occasion during Pregnancy	Paternal Drinking Problem ^e^
Verbal ability ^a^ (log)	Partial *r*	**−0.134**	**−0.148**	**−0.140**	−0.083	−0.099	−0.005
	*p ^f^*	0.007	0.003	0.005	0.096	0.047	0.927
	*n*	397	396	398	399	399	344
Non-verbal ability ^b^ (log)	Partial *r*	**−0.151**	**−0.145**	**−0.146**	−0.110	−0.087	−0.055
	*p ^f^*	0.002	0.004	0.003	0.027	0.081	0.304
	*n*	399	397	399	400	400	345
WISC-IV Digit Span ^c^	Partial *r*	**−0.151**	**−0.164**	**−0.168**	−0.098	−0.106	−0.069
	*p ^f^*	0.002	0.001	0.001	0.048	0.032	0.198
	*n*	397	397	399	400	400	345
Behavior problems ^d^ (sqrt)	Partial *r*	**0.222**	**0.178**	**0.176**	**0.191**	**0.148**	0.038
	*p ^f^*	<0.001	<0.001	<0.001	<0.001	0.003	0.175
	*n*	402	401	403	404	404	348
Inattention problems ^d^ (sqrt)	Partial *r*	**0.219**	**0.165**	**0.166**	**0.175**	**0.149**	0.026
	*p ^f^*	<0.001	0.001	0.001	<0.001	0.002	0.624
	*n*	401	400	402	403	403	348
Head circumference	Partial *r*	**−0.224**	**−0.256**	**−0.255**	**−0.206**	**−0.217**	−0.028
	*p ^f^*	<0.001	<0.001	<0.001	<0.001	<0.001	0.596
	*n*	410	409	411	412	412	355
Dysmorphology score	Partial *r*	**0.186**	**0.193**	**0.215**	**0.168**	**0.186**	−0.001
	*p ^f^*	<0.001	<0.001	<0.001	0.001	<0.001	0.993
	*n*	408	407	409	412	410	353

^1^ All scores standardized for age of child at time of testing. ^a^ Tests of the Reception of Grammar (TROG); ^b^ Raven Coloured Progressive Matrices; ^c^ Wechsler Intelligence Scales for Children, Third Edition; ^d^ Teacher Report Form ; ^e^ Also adjusted for Mothers’ Drinks per Week (log); ^f^ Bonferroni adjusted value *p* < 0.012. Bold numbers indicate significance at the Bonferroni adjusted value.

**Table 6:** Prevalence rates (per 1000) of individual diagnoses within fetal alcohol spectrum disorders (FASD) and total FASD by three methods of estimation from a second sample in South African communities.

	Oversample of Children ≤25th Centile on Height, Weight, or OFC			Random Sample Rate of FASD Diagnoses and Estimated Cases in the Non-Consented Children					Combined Rate from Cases in Consented Sample (*n* = 1088) and Estimated Cases in Non-Consented Sample (*n* = 365)		
	(a) Total Cases Diagnosed *n*	Low Estimated Rate: School Enrollment Rate ^1^ (*n* = 1448)	Consented Student Rate ^2^ (*n* = 1088)	Cases Found among Randomly-Selected Controls *n*	Proportion of FASD Cases in Random Sample (*n* = 276)	(b) Estimated Cases in Non-Consented Sample (*n* = 365)	Rate of FASD from Random Sample Only ^3^	95% CI	(a + b) Total Estimated Cases (*n* = 399)	High Estimated Rate: Estimated Rate for All Enrolled Students ^4^	95% CI
FAS	129	89.1	118.6	44	0.1594	58	159.4	116.2 to 202.6	187	129.1	111.8 to 146.4
PFAS	100	69.1	91.9	28	0.1014	37	101.4	65.8 to 137.1	137	94.6	79.5 to 109.7
ARND	55	37.9	50.6	15	0.0543	20	54.3	27.6 to 81.1	75	51.8	40.4 to 63.2
Total FASD	284	196.1	261.0	87	0.3152	115	315.2	260.4 to 370.0	399	275.5	252.5 to 298.6

^1^ Denominator is all children attending first grade in local schools. Rate per 1000 based on entire enrollment in 1st grade classrooms (*n* = 1448). ^2^ Denominator is the total number of children with consent to participate. Rate per 1000 based on the sample consented and screened (*n* = 1088). ^3^ Calculated as the FASD cases diagnosed from the randomly-selected control candidates (numerator) over total number of randomly-selected children ×1000. ^4^ Rate per 1000 children calculated from FASD cases diagnosed in the consented sample (a) added to the estimated cases in the non-consented sample utilizing the proportional diagnostic distribution of FASD cases among randomly-selected children (b), and divided by all 1st grade children enrolled in the schools (*n* = 1448).

**Table 7:** Comparison of two samples in rural communities of the western cape province of South Africa (rates per 1000 first grade children).

	Sample 1 (Initiated in 2009) [33]	Sample 2 (Initiated in 2011)	Sample 1 Case Ratio: Cases per FAS Case	Sample 2 Case Ratio: Cases per FAS Case
FAS	92.7–127.0	89.1–129.1	-	-
PFAS	58.4–86.2	69.1–94.6	0.68	0.73
ARND	31.6–45.6	37.9–51.8	0.36	0.40
Total FASD	182.7–258.9	196.1–275.5	1.04	1.13
